# Unmet Needs for Community-Based Rehabilitation the First 12 Months Postinjury in a Traumatic Brain Injury Population with Moderate-to-Severe Trauma—a Longitudinal Cohort Study

**DOI:** 10.1016/j.arrct.2026.100619

**Published:** 2026-03-27

**Authors:** Helene Lundgaard Soberg, Mari Storli Rasmussen, Håkon Øgreid Moksnes, Christoph Schäfer, Audny Anke, Nada Andelic, Torgeir Hellstrøm

**Affiliations:** a Faculty of Health Sciences, Oslo Metropolitan University, OsloMet, Oslo.; b Department of Physical Medicine and Rehabilitation, Oslo University Hospital, Oslo.; c Department of Rehabilitation, University Hospital of North Norway, Tromsø.; d Faculty of Health Sciences, Department of Clinical Medicine, UiT The Arctic University of Norway, Tromsø.; e Research Centre for Habilitation and Rehabilitation Models and Services (CHARM), Institute of Health and Society, Faculty of Medicine, University of Oslo, Oslo, Norway.

**Keywords:** Multiple trauma, Personal health, Rehabilitation, Rehabilitation needs, services, Traumatic brain injuries

## Abstract

**Objective:**

To describe and explore factors related to unmet needs and differences in urban vs rural centrality of community-based rehabilitation in patients with moderate-to-severe traumatic injuries with TBI at 12 months postinjury.

**Design:**

A 12-month follow-up study of rehabilitation needs in a longitudinal multicenter cohort study Rehabilitation Needs, Service Provision, and Costs in the First Year after Traumatic Injuries.

**Setting:**

Two reginal trauma centers and the patients’ municipality of residence.

**Participants:**

Patients with moderate-to-severe trauma with a traumatic brain injury with age≥18 years, a New Injury Severity Score>9, an Abbreviated Injury – Head score≥2, admission to the trauma centers within 72 hours, and a hospital stay of at least 2 days. N=235 community dwelling patients (mean age 51.9y; 77.4% men) were included.

**Interventions:**

Not applicable.

**Main Outcome Measures:**

Unmet rehabilitation needs at 12 months on the Needs and Provision Complexity Scale. We hypothesized that injury severity and urban vs rural centrality of residence would predict unmet rehabilitation needs at 12 months postinjury.

**Results:**

42.1% reported unmet rehabilitation needs on the Needs and Provision Complexity Scale Rehabilitation subscale at 12 months postinjury. Median unmet rehabilitation needs was 2.0 (interquartile range 1-3). Mean Glasgow Outcome Scale Extended (GOSE) was 6.2 (SD, 1.5).

A logistic regression model that included demographic and injury-related variables, urban vs rural centrality of residence and global functioning on the GOSE found that significant predictors of unmet rehabilitation needs were living in less central municipalities (odds ratio [OR], 2.18), AIS-Head≥3 (OR, 3.35), and lower GOSE scores at 6 months (OR, 1.60).

**Conclusions:**

Unmet rehabilitation needs are common after moderate-to-severe trauma with TBI at 12 months postinjury. Particular attention is required for individuals with more severe TBI and those in less central municipalities, because they face higher risk of unmet rehabilitation needs.

## Highlights

• The Needs and Provision Complexity Scale captures unmet rehabilitation needs.
• Patients with traumatic injuries with brain injury need long-term rehabilitation.
• Predictors of unmet rehabilitation needs at 12 months were:
• Living in less central areas, brain injury severity and low function at 6 months.

Rehabilitation after severe injuries requires structured, coordinated services across administrative levels—from highly specialized national or regional services to community-based services.^1^, ^2^, ^3^ The importance of addressing the rehabilitation needs of injured individuals is emphasized in the World Health Organization Rehabilitation 2030 initiative, which calls for strengthened rehabilitation services with focus on improving access and integrating care at the individual level.^4^^,^^5^

Sustaining severe multiple injuries including traumatic brain injury (TBI), require long-term rehabilitation. Many patients report persistent functional limitations and difficulty returning to work years postinjury.^6^, ^7^, ^8^, ^9^, ^10^ Problems in cognitive functioning and fatigue are common sequelae after TBI.^11^^,^^12^ In individuals with multiple injuries, the presence of a TBI may pose additional challenges in meeting rehabilitation needs, particularly within the long-term municipal service provision.^13^

Rehabilitation efforts should extend beyond acute and subacute care.^9^^,^^14^, ^15^, ^16^ A discrepancy between rehabilitation needs and service provision has been documented, with many trauma and TBI survivors experiencing unmet needs after hospital discharge.^17^, ^18^, ^19^ In a multiple trauma population where 72% had severe to profound injuries nearly all patients reported needs for functional rehabilitation, sociovocational support and community integration services when interviewed 2-4 years postinjury.^20^ Conversely, other studies have demonstrated geographic disparities in trauma patients with TBI in service provision with more unmet needs in less densely populated areas compared with urban areas.^21^, ^22^, ^23^

Despite increasing recognition of needs for long-term rehabilitation services after severe injuries and TBI, few studies have examined community-based rehabilitation needs—defined as the specific services required by individuals with moderate-to-severe trauma with TBI—and the gaps between needs and actual service provision.^24^ This study contributes to the existing literature by incorporating health care, social care and rehabilitation needs, including unmet needs, using the Needs and Provision Complexity Scale (NPCS), a tool specifically developed to assess the extent of needs in individuals with neurologic disabilities.^25^

We aimed to describe unmet needs for community-based rehabilitation in patients with TBI and moderate-to-severe traumatic injuries at 12-months postinjury. Additionally, we explored associations between sociodemographic factors, residential centrality, clinical characteristics, and unmet needs, hypothesizing that injury severity and centrality of residence would predict unmet rehabilitation needs.

## Methods

### Design, participants, and procedure

**Fig 1:**
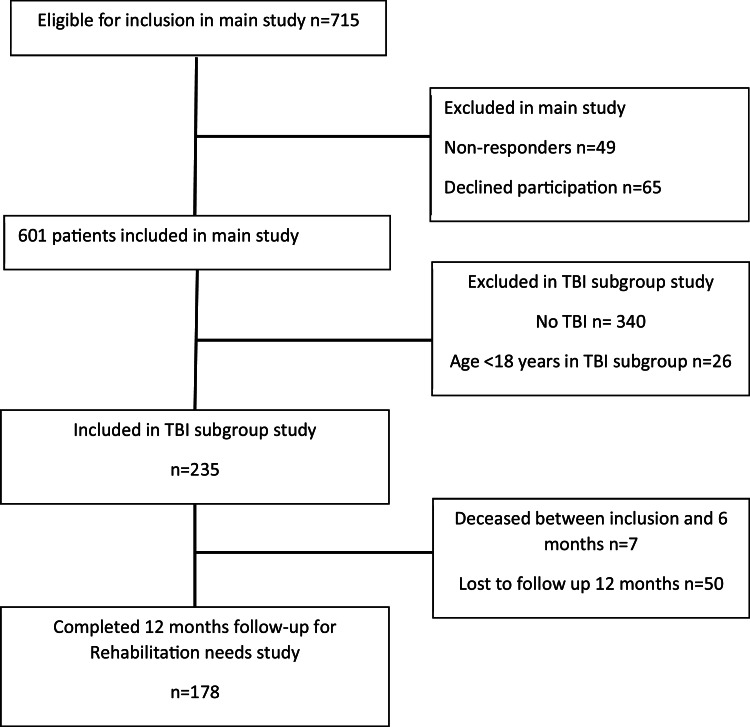
Flow chart from eligible patients in the main study to the rehabilitation needs study of the trauma patients with TBI included in the 12 month follow-up study.

Abbreviations: TBI, traumatic brain injury.

This is a subgroup study that included 6- and 12-month follow-ups, derived from a larger prospective longitudinal multicenter study named Rehabilitation Needs, Service Provision, and Costs in the First Year after Traumatic Injuries of a 1-year cohort of patients who sustained moderate-to-severe trauma.^26^^,^^27^ The current study population comprises patients who sustained a TBI among their injuries and were admitted in 2020 to regional trauma centers at Oslo University Hospital or the University Hospital of North Norway. Inclusion criteria were patients aged ≥18 years with a New Injury Severity Score (NISS)>9 and a TBI with an Abbreviated Injury Scale (AIS)-Head score≥2.^28^^,^^29^ According to the NICE guidelines patients with ISS>9 admitted to trauma units should be assessed for rehabilitation needs.^3^ We applied the NISS for describing the overall injury severity in this study, as it captures the extent of injuries independent of body region. Eligible patients were admitted within 72 hours postinjury and had a hospital stay of at least 2 days. Exclusion criteria included non-Norwegian residency and non-Norwegian/English speakers. See flow chart in figure 1.

The inclusion procedure is detailed in a previous publication.^30^ Briefly, eligible patients were identified in trauma meetings, hospitalization lists, and medical record reviews. Patients fulfilling the inclusion criteria or their caregiver received study information and provided written informed consent prior to participation.

The STROBE guidelines were applied. The study was approved by the Regional Committee for Medical and Health Research Ethics (# 31676). The study was conducted in accordance with the principles of the Declaration of Helsinki.

### Variables and outcome measures

Demographic information included age, sex (male/female), marital status (single/widowed; married/cohabiting), education level (low ≤13y; high ≥14y), work status (working/student; retired; unemployed). Injury-related data were injury mechanism (falls, transport accidents, violence, other). In the AIS, injuries in different body regions are assigned a severity code ranging from 1 (minor) to 6 (maximal, currently untreatable).^29^ Patients with an AIS-Head score≥2 were included in this study as an AIS-Head score of 1 describes minor head injury. The NISS is based on the summed square of the 3 most severe injuries according to the AIS, independent of body region. A NISS of 10-15 is categorized as a moderate injury, 16-25 as a severe injury, and 26-75 as a profound injury.^17^ Other injury-related variables were length of stay in acute hospital (LOS) (d), and discharge destination (home; local hospital; rehabilitation institution, nursing home/other). The American Anesthesiologists physical status classification system (ASA) describes preinjury comorbidities, in this study, on a 4-point scale from healthy to severe systemic disease that poses a constant threat to life.^31^ The scores 5 moribound and 6 brain dead were not present in our study. The ASA was dichotomized into healthy/mild systemic disease (ASA 1) and Severe systemic disease (ASA≥2). Geographic centrality of the municipality of residency was classified according to the Norwegian Centrality Index (NCI) on a scale from 1 (high centrality) to 6 (low centrality).^32^ The NCI was dichotomized into central (NCI 1-2) and less central (NCI 3-6).

Postinjury functioning was assessed by the Glasgow Outcome Scale Extended (GOSE) through structured patient interviews conducted by telephone at 6- and 12-months postinjury.^33^ The scale ranges from 1 (dead) to 8 (fully returned to normal life). The scores are categorized as Severe disability (3-4), Moderate disability (5-6), and Good recovery (7-8).^34^ In the current study, the GOSE scores at 6 months were applied in the regression analysis.

The Needs and Provision Complexity Scale (NPCS) assesses met and unmet community-based health service needs and has demonstrated good to excellent interrater and test–retest reliability in a Norwegian TBI population.^25^^,^^35^ The NPCS includes 15 items across 2 domains—Health and Personal Care (0-25 points) with subscales for Health care (0-6), Personal care (0-10), and Rehabilitation (0-9 points), and social care and support (0-25 points) with the subscales social and family support (0-13) and Environment (0-12). The clinician-rated NPCS-Needs and the patient-reported NPCS-Gets both yield total scores from 0-50, with higher scores indicating greater needs. The rehabilitation subscale comprises therapy disciplines, therapy intensity, and vocational/educational support needs. Unmet needs on the NPSC and its subscales at 12 months are categorized according to Turner-Stokes et al,^25^ with differences ≥1 categorized as unmet needs. The primary outcome was unmet rehabilitation needs at 12 months, calculated by subtracting clinicians’ NPCS-Needs scores at 6 months from patients’ NPCS-Gets scores obtained at 12-month telephone interviews, followed by dichotomization. Clinician ratings at 6 months were based on specialist rehabilitation expertise and assessments incorporating acute-phase medical record information and patient-reported symptoms and functioning at 6 months.

### Statistical analysis

Descriptive data are presented as mean (SD) or median (interquartile range [IQR]), and categorical variables as frequencies and relative proportions (%). Missing data on participant characteristics were obtained at the 6-month follow-up. Group differences regarding lost to follow-up were analyzed.

The primary outcome and dependent variable was dichotomized into met (≤0) and unmet (≥1) needs on the NPCS rehabilitation subscale at 12 months.

Univariate logistic regression analyses assessed associations between rehabilitation needs at 12 months and the candidate independent variables personal factors (age, sex: male/female; marital status: married/cohabiting; yes/no, level of education: low/high [</≥13 years], centrality of residence [central/less central], preinjury comorbidities on the ASA [no/yes]) and injury-related factors (Glasgow Coma Scale [GCS] score,^36^ AIS-Head [score</≥3], the NISS, LOS, and GOSE at 6 months postinjury). In addition we adjusted for sex, age, and education. Multicollinearity was checked, and variables with *r*≥0.70 were not entered together. The final model included 170 patients. Results are reported as odds ratios [ORs] (95% CI), and model fit was evaluated using Hosmer–Lemeshow goodness-of-fit and Nagelkerke *R*² to ensure no violation of the of the regression model. A 2-sided 5% significance level was applied. Analyses were conducted using IBM SPSS Statistics v29 (IBM Corp).

## Results

The mean age of the 235 trauma patients with TBI was 51.9 years (SD, 17.6), and 77.4% were men. More than half (55.7 %) resided in central municipalities. The mean NISS was 31.3 (SD, 14.3) with 26.4% classified as having severe injuries and 60.9% as profound. Most patients (84.3%) sustained serious, severe to critical brain injuries (AIS-Head ≥3). On average, the patients sustained 7.1 (SD, 3.9) injuries, and 99% had multiple injuries. The median GCS score at admission was 12, with 33% of the patients scoring ≤12 points. Table 1 presents demographic and injury-related data. There were no significant differences in patients lost to follow-up from 6-12 months regarding age, sex, education, AIS-Head severity or centrality of residence. Among patients available at the 12-month follow-up, 4.5% were excluded from the regression analysis because of missing data.

**Table 1:** Demographic, injury-related and discharge destination characteristics of the trauma patients with TBI and an AIS-Head score≥2, presented as n (%), mean (SD), or median (IQR).

Demographic Variables n=235	
Sex (male)	182 (77%)
Age, mean (SD)	51.9 (17.6)
Marital status	
Single/widowed	98 (41.7%)
Married/cohabiting	137 (58.3%)
Education (n=219)	
High school (low ≤13y)	122 (55.7%)
University (high ≥14y)	97 (44.3%)
Work status (n=231)	
Working/Student	140 (60.6%)
Retired	48 (20.8%)
Unemployed	43 (18.6%)
Centrality of municipality of residence	
Central	131 (55.7%)
Less central	104 (44.3%)
Comorbidities ASA* (n=235)	
Healthy/Mild systemic disease	199 (84.7%)
Severe systemic disease	36 (15.3%)
**Injury-related variables (n=227)**	
Injury mechanism	
Fall	113 (48.1%)
Traffic	80 (34%)
Sport	6 (2.6%)
Other	28 (11.9%)
GCS†, median (IQR)	12 (9-15)
GCS category (n=234)	
Mild (15-13)	157 (67.1%)
Moderate (12-9)	22 (9.4%)
Severe (8-3)	55 (23.5%)
NISS‡, mean (SD)	31.3 (14.3)
AIS§-Head≥3 (serious-critical injury)	198 (84.3%)
Number of injuries, mean (SD)	7.1 (3.9)
LOS‖ (d), median (IQR)	8 (3-18)
Discharge destination	
Home	55 (23.4%)
Local hospital	92 (39.1%)
Rehabilitation institution	81 (34.5%)
Nursing home/other	7 (3%)

Abbreviation: IQR, interquartile range.

⁎ American Anesthesiologists Physical Status Classification System.

† Glasgow Coma Scale.

‡ NISS.

§ Abbreviated Injury Scale.

‖ Length of stay.

Global functioning, measured by the GOSE, corresponded to moderate disability at both 6 and 12 months with mean scores of 5.8 (SD, 1.6) and 6.1 (SD, 1.7) points, respectively. The global functional level, as assessed using the GOSE mean score, did not change substantially from 6 to 12 months. At 6 months 54.7% had moderate disability and 13.3% severe disability. At 12 months 43.8% had moderate disability, and 9.6% severe disability.

Among the 178 (76%) patients who provided rehabilitation information at 12 months the number of unmet needs on the NPCS rehabilitation scale was median 2 (IQR, 1-3). 49.4% had unmet needs per the NPCS total score, and 42.1% on the rehabilitation subscale which comprises therapy disciplines, therapy intensity, and vocational/educational support. The median number of therapy disciplines was 1 (IQR, 0-1; range 0-3), with 45% reporting no services. Therapy intensity had a median score of 1 (IQR, 0-2; range 0-3), with 45.5% receiving none. Vocational/educational support had a median of 0 (IQR, 0-0; range 0-3) contacts. Table 2 details unmet needs across NPCS domains and subscales.

**Table 2:** The proportion n (%) of patients with unmet needs on the Needs and Provision Complexity Scale total scale and subscales at 12 months postinjury.

NPCS Main Domains Unmet needs 12 mo (no.)	No. (%) With Unmet Needs	Subscales	No. (%) With Unmet Needs
Health and personal care (n=178)	78 (44.1)	Health care	40 (22.5)
		Personal care	17 (9.6)
		**Rehabilitation**	**75 (42.1)**
Social care and support (n=176)	44 (24.7)	Social and family support	39 (22.2)
		Environment	9 (5.1)
NPCS Unmet needs total score (n=176)	87 (49.4)		

Abbreviation: NPCS, Needs and Provision Complexity Scale.

The overall logistic regression model predicting unmet rehabilitation needs was statistically significant (chi-square [*df*=10]=36.86, *P*<.001), and explained 26% of the variance (Nagelkerke *R*^2^) (table 3). The model correctly classified 69% of cases (not shown in table). Significant predictors included residing in a less central municipality, AIS-Head≥3, and lower GOSE at 6 months. Demographics, comorbidities, and the initial injury parameters GCS and LOS were not significant. Adjusted odds of having unmet rehabilitation needs were 2.2 times higher in less central areas, 4.0 times higher with an AIS-Head≥3, and increased 1.6 times per category of worse GOSE at 6 months.

**Table 3:** Results of the univariate and multiple logistic regression analyses of factors associated with unmet rehabilitation needs at 12 months post-TBI on the Needs and Provision Complexity Scale Rehabilitation subscale.

Characteristics	Univariate Logistic Regression		Multiple Logistic Regression	
	OR (95% CI)	*P* Value	OR (95% CI)	*P* Value
Sex (male/female)	1.23 (0.60-2.52)	.565	1.14 (0.49-2.69)	.754
Age (y)	1.01 (0.99-1.03)	.231	1.00 (0.98-1.02)	.821
Education low/high	1.00 (0.98-1.01)	.768	0.99 (0.48-2.04)	.978
Centrality Index Central/less central	2.22 (1.21-4.09)	.010	2.18 (1.06-4.50)	**.035**
ASA no/yes	.49 (0.98-6.36)	.056	2.41 (0.74-7.84)	.143
GCS at admission	0.92 (0.86-0.99)	.028	1.03 (0.93-1.14)	.619
AIS-head **2**/≥3	4.03 (1.45-11.15)	.007	3.35 (1.13-9.88)	**.029**
LOS (d)	1.03 (1.01-1.05)	.006	1.01 (0.98-1.04)	.482
Number of injuries	1.10 (1.01-1.20)	.024	1.04 (0.93-1.17)	.470
GOSE at 6 months postinjury	0.55 (0.42-0.71)	<.001	0.64 (047-0.86)	**.004**
Wald Nagelkerke *R*^2^ Cox&Snell *R*^2^ Hosmer–Lemeshow		3.95 0.262 0.195 11.143		*P*=.047 *P*=.194

N=170.

Abbreviations: AIS, Abbreviated Injury Scale; ASA, American Anesthesiologists Physical Status Classification System CI, confidence interval; GCS, Glasgow Coma Scale; GOSE, Glasgow Outcome Scale Extended; LOS, length of stay; OR, odds ratio.

## Discussion

This study shows that a large proportion of patients with moderate-to-severe trauma including TBI had unmet rehabilitation needs at 12 months postinjury. Factors associated with these unmet needs include more severe TBI, lower global functioning at 6 months postinjury and residing in less central areas.

Sustaining multiple injuries require long-term specialized rehabilitation, and guidelines emphasize the importance of a continuous rehabilitation trajectory.^1^, ^2^, ^3^ In this study about one-third of patients were discharged directly to a rehabilitation institution from acute care. Others were discharged to local hospitals (39%), possibly awaiting specialized rehabilitation,^37^ or directly home (23%) where municipal health and rehabilitation services assume responsibility. Patient representatives in a focus group study emphasized that professionals with specialized competence are needed to address specific rehabilitation needs, contrasting with the generalist services typically available in municipalities.^38^

Nearly all participants in this study had sustained multiple injuries. Previous research has demonstrated that people with TBI frequently experience fragmented rehabilitation services.^39^^,^^40^ Individuals with multiple injuries are often referred to the specialty perceived as addressing the most severe injury, potentially leaving cognitive and neurologic rehabilitation needs unmet.^41^ Findings from the present study corroborate this concern: numerous multitrauma patients with TBI reported unmet rehabilitation needs. According to the NPCS-Gets scores, participants received limited therapy between 6 and 12 months postinjury, particularly regarding therapy intensity and vocational/educational support.

Health and rehabilitation services are key components of universal health coverage according to the World Health Organization and should be equally accessible regardless of living conditions or location.^42^^,^^43^ However, this study found that residing in less central municipalities increased the likelihood of unmet rehabilitation needs by a factor of 2.2. This finding aligns with prior research that identified limited access to appropriate services particularly in rural regions.^24^ Contributing factors include limited transportation options, shortages of qualified staff and rehabilitation professionals, and insufficient availability of cognitive and mental health rehabilitation services. Furthermore, long-term rehabilitation receives less governmental support compared to earlier rehabilitation phases across Europe.^24^ In contrast, an Australian study on patients with TBI by Hennessy et al^44^ reported that remoteness was not associated with unmet rehabilitation needs.

The TBI population in the current study constituted 44% of the patients aged 18 years and older in the larger multicenter project.^30^ The high mean NISS score shows that the study population is predominantly composed of patients with severe to profound injuries. Moreover, admission GCS and the AIS-Head scores suggest that many patients were likely to require cognitive rehabilitation services. Although both variables could be included in the multiple regression model, only AIS-Head remained a statistically significant predictor of unmet rehabilitation needs. Level of consciousness at admission, as measured by GCS, can be influenced by factors such as blood loss, intoxication, and prehospital sedation,^45^ whereas the AIS-Head score provides a more valid indicator of brain injury severity.^46^

More than 50% of the patients were still categorized as having moderate-to-severe disability on the GOSE at 12 months and in need of rehabilitation services as assessed by the NPCS. Furthermore, the functional level on the GOSE at 6 months predicted unmet rehabilitation needs at 12 months. This aligns with previous research, showing that while GOSE scores generally improve during the first year after injury, full recovery is often not achieved.^41^

TBI and multiple trauma frequently affect individuals of working age, making postinjury work participation a critical issue.^47^ The mean age of 52 years in the current study suggests that return to activities, education and work are key rehabilitation goals.^48^, ^49^, ^50^ Schäfer et al^6^ reported that about half of patients with TBI as the dominating injury had not fully returned to work at 12 months, underscoring the need for improved vocational support. Rehabilitation should be individualized through shared decision-making and multiprofessional services.^5^^,^^51^ However, physiotherapy remains the most common service after trauma, whereas mental and cognitive rehabilitation is often insufficient.^18^^,^^52^ Although the Norwegian Welfare and Labour Administration provides follow-up for individuals on sick leave, stronger collaboration between health care, educational, and social welfare systems is needed after trauma. A cognitive rehabilitation and supported-employment model has shown benefits for early return to work in mild-to-moderate TBI.^53^ This study highlights the need for similar collaboration for TBI patients within the multiple trauma population.

### Study limitations

Strengths of this study include its prospective design, use of hospital trauma registry data to validate injury severity scores, low proportion of missing data, and an acceptable loss to follow-up rate (21%).

A potential limitation is that patient inclusion occurred during the first wave of the COVID-19 pandemic, which may have influenced rehabilitation services. However, the health care system remained relatively adaptable,^54^ and rapid telehealth expansion helped ensure continuity of ambulatory care. Further, rehabilitation specialists may have overestimated needs, possibly because of underestimating functional improvements between 6 and 12 months. Their estimates were based on patient-reported impairments and the assumption that appropriate services were available; where services were lacking, patients continued to report unmet needs.^25^ Recall bias may also have influenced patients’ reports, as individuals with TBI might struggle to accurately recall services received during this period. Another limitation of the study is that profession specific information regarding unmet rehabilitation needs was not accessible.

Nonetheless, these findings align with previous research on long-term neurologic disabilities, which similarly identified substantial gaps between service provision and patient needs during the first year after discharge from inpatient neurorehabilitation.^55^

## Conclusions

Unmet rehabilitation needs are common after moderate-to-severe trauma with TBI at 12 months. Addressing rehabilitation needs systematically should be an integral part of the rehabilitation process. Particular attention is required for individuals with more severe TBI and those in less central municipalities, as they face higher risk of unmet rehabilitation needs.

## Disclosure

The investigators have no financial or nonfinancial disclosures to make in relation to this project.
